# Edematous Bullae: An Atypical Presentation of Reperfusion Injury. A Discussion of Ischemic-reperfusion Injury and Presentation of an Atypical Case

**DOI:** 10.7759/cureus.5376

**Published:** 2019-08-13

**Authors:** Manick Saran, Sean Malarkey

**Affiliations:** 1 Miscellaneous, Lake Erie College of Osteopathic Medicine, Erie, USA; 2 Vascular Surgery, Allegheny Health Network, Pittsburgh, USA

**Keywords:** ischemia-reperfusion, vesicles and bullae, peripheral artery disease, femoropopliteal bypass

## Abstract

There is vast literature on the topic of ischemia-reperfusion injury. A summative discussion of the complex pathogenicity will aid practicing physicians in diagnosis and management. We offer a review of this literature as well as a discussion on a rare case of tense edematous bullae as a presentation of ischemia-reperfusion injury. A 65-year-old male underwent a right femoropopliteal bypass for rest pain that had not improved after iliac stent placement. He presented three days after discharge with blistering lesions on the reperfused limb that resembled bullous pemphigoid. This case describes the variability in the presentation of reperfusion injury, as well as the necessity to educate those managing atypical presentations of reperfusion injury.

## Introduction

Patients with peripheral vascular disease can become severely debilitated in cases of severe ischemic rest pain due to decreased blood flow in the extremities. Modern medicine has provided surgeons a way to improve the outcome of these cases by the revascularization of the extremity. Revascularization of the extremity does not only improve symptoms such as ischemic rest pain, but also improves energy, pain, emotional reactions, sleep, paid employment, and hobbies [1]. This indicates that the overall quality of life improves after revascularization. However, revascularization has its own consequences. One of the most unfortunate consequences is reperfusion injury. Here, we present an atypical case of reperfusion injury and discuss the importance of recognizing the signs and symptoms associated with it.

## Case presentation

A 65-year-old Caucasian male presented to the office with episodes of paresthesias in his right foot. His medical history included hypertension and 45 pack-years of smoking. He complained of paresthesias and pain in his right foot for the previous three weeks. The foot pain had reached the point at which he could no longer complete his daily occupation as a custodian. Physical exam revealed the lack of a palpable pulse on either the dorsalis pedis artery or the posterior tibial artery on the right side. Femoral pulses were 2+ on both the right and left sides. The initial ankle-brachial index (ABI) measurement in the office was approximately 0.3. Further angiography showed occlusion of the above-knee popliteal artery and subsequent reconstitution of flow into the below-knee popliteal artery, as well as multiple stenoses in the superficial femoral artery. Biphasic waveforms were present in the right common femoral artery, profundal artery, and superficial femoral artery proximally. The right mid-superficial femoral artery, posterior tibial artery, and dorsalis pedis waveforms were monophasic. At that time, he was started on Atorvastatin and scheduled for a right-sided femoral angiography with possible stenting. During the procedure, his right common iliac artery was found to have about 90% stenosis with both external and internal iliac artery patent, with no disease. The findings in the right leg during the procedure showed both the right deep femoral artery and superficial femoral artery patent. The distal right superficial femoral artery, however, showed 80% stenosis in multiple segments. Both the popliteal artery behind the knee and the anterior tibial artery at the level of the right ankle were completely occluded. His right iliac stenosis was treated with balloon-expandable stent placement, with the subsequent resolution of common iliac artery disease shown through angiography postop.

Two weeks after the procedure, he presented back to the office with no improvement in symptoms and newly developed ischemic rest pain and hyperalgesia. He stated that he could barely walk due to the pain. He was started on intravenous heparin with plans for a below-the-knee bypass procedure. During the bypass, a right common femoral endarterectomy with bovine patch angioplasty was performed. The bypass was done using a Propaten graft (Gore Medical, Arizona, US), as preoperative vein mapping found the greater saphenous vein to be a poor conduit. At the conclusion of the procedure, both the popliteal and dorsalis pedis pulses were palpable. His postoperative course was uneventful, and he was subsequently discharged four days after his surgery.

**Figure 1:**
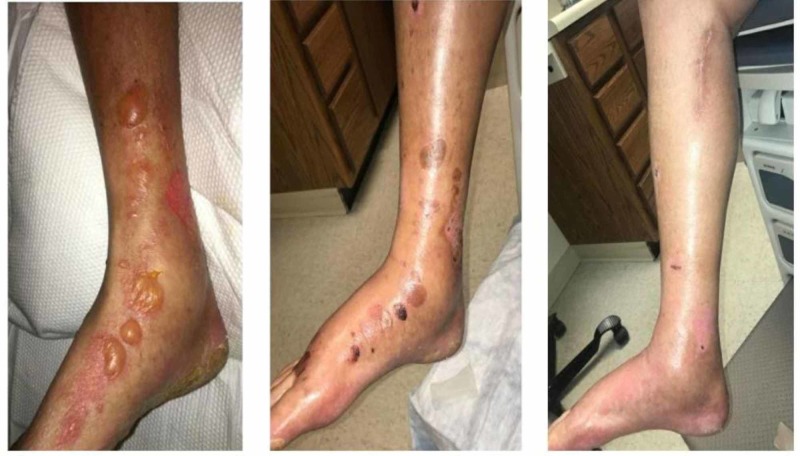
Edematous lesions Pane 1, Initial presentation; Pane 2, One-week follow-up; Pane 3, Two-week follow-up.

Two days after discharge, he presented to the emergency department (ED) with multiple blisters on the dorsum of his right foot scattered through the lower leg (see Figure 1, Pane 1). Signs of dehiscence or cellulitis were not present. He stated they had started shortly after he was discharged. They measured 1 cm and were described as tense with fluid. His labs at the time of presentation were significant for a C-reactive protein (CRP) of 3, white blood cell (WBC) of 15,000 per microliter, with an absolute neutrophil count of 13,000 per microliter. The differential diagnoses of the presentation included edematous lesions, bullous impetigo, and bullous pemphigoid. The incision site was clean, dry, and intact with no evidence of cellulitis. The patient was treated for suspected bullous impetigo and given topical mupirocin. However, the lesions did not improve. The patient did not see any resolution of the lesions until topical clobetasol ointment was added to the regimen. The patient was discharged three days later on a topical clobetasol ointment.

The first follow-up appointment after discharge was almost a week later, in which the patient stated he had been continuing with his ointment with gauze dressings. At that time, his leg appeared to be resolving (see Figure 1, Pane 2). The patient returned a week later for a two-week follow-up and the lesions had almost completely resolved (See Figure 1, Pane 3). At that time, the main complaint of ischemic pain had dissipated and the residual swelling and bullae from the operation had decreased.

## Discussion

Peripheral arterial disease (PAD) currently affects roughly 6% of people over the age of 40 [2], with common underlying risk factors, consisting of diabetes, hypertension, tobacco abuse, hyperlipidemia, and obesity, in our aging population. With better screening and management of these risk factors, PAD has been on the decline as demonstrated by Berger et al. who showed the prevalence of PAD in the US to be at around 3.5% [3]. Patients often present with typical signs of PAD such as intermittent claudication and ulceration of the extremity, with those on the worst side of the spectrum presenting with ischemic rest pain. Ischemic rest pain results from the severe decrease in limb perfusion typically presenting itself when the patient elevates their lower extremity such as at bedtime. Patients will often feel relief by hanging their feet off the edge of the bed to restore what little blood flow is left to the extremity.

Those presenting with the clinical features listed above, combined with appropriate risk factors, will then undergo an ankle-brachial index (ABI) to confirm the diagnosis of PAD. According to American Heart Association/American College of Cardiology (AHA/ACC) guidelines on the management of lower extremity peripheral artery disease, those with possible PAD and ABI <0.9 are considered abnormal [4]. Duplex ultrasound may also be used in conjunction with ABI to get a better idea of the degree and location of the stenotic vessel. The management of PAD can range from simple lifestyle modifications, including smoking cessation, diet, and exercise, to surgical management. The medical management of PAD includes the initiation of statin therapy, antithrombotic therapy, and glycemic control.

Surgical management, including revascularization, is indicated in those who continue to have symptoms despite lifestyle modification and medical management and those who present with limb-threatening ischemia. According to an article by DeWeese et al., when discussing lower extremity revascularization, complications include bleeding, edema, skin necrosis, infection, bypass thrombosis, pseudoaneurysm formation, pulmonary embolism, stroke, myocardial infarction (MI), and death [5]. Interestingly, post-revascularization edema presented most commonly in those patients undergoing a femoropopliteal bypass [6].

Reperfusion injury is a complex and often dramatic acute response to the revascularization of a previously ischemic limb. Ischemic reperfusion injury is a counterintuitive concept, as reperfusion to the ischemic limb should be of utmost importance. However, when reperfusion occurs, the limb may become more damaged than before the procedure. A study done by Parks et al. showed that the histologically identified mucosal injury was more severe in tissue that was exposed to reperfusion after three hours of ischemia as compared to the tissue that underwent four hours of ischemia alone [7]. Several underlying biochemical changes in the vascular wall of ischemic tissue allow for the development of reperfusion injury. The biochemical and molecular changes include alterations to mitochondrial and cellular function, the production of reactive oxygen species, and inflammatory mediators that play a role upon reperfusion of the ischemic tissue.

To understand the pathophysiology behind an ischemia-reperfusion injury, a superficial understanding of biochemical processes must be understood. It is well-known that cells undergo oxidative phosphorylation to produce the energy required for basic cellular processes. In the presence of prolonged anoxia, these oxidative phosphorylation reactions fail to produce energy, resulting in the failure of cell membrane pumps dependent on adenosine triphosphate (ATP). The failure of these pumps allows for the accumulation of sodium and, subsequently, water within cells. Calcium accumulates intracellularly as well, resulting in cellular lipid and protein damage.

In 1981, Granger et al. demonstrated for the first time the central role of free radical formation in reperfusion injury. The accumulation of hypoxanthine in cells undergoes oxidation by xanthine oxidase, an enzyme increased in times of ischemia [8], to produce a superoxide free radical as a by-product. Granger et al. discovered that the administration superoxide dismutase (SOD), an inhibitor of superoxide, just prior to reperfusion prevented injuries caused by reperfusion [9]. This superoxide ion can damage the cell in a variety of ways, including the oxidation of biomolecules, as well as the inactivation of intracellular enzymes [10].

Central to the development of edema, as in our patient, are inflammatory mediators that are typically present in infectious processes. The process of leukocyte extravasation is well-known in the literature and includes the steps of margination, adhesion, transmigration, and migration. Here, the role of this process in ischemia-reperfusion is discussed. The initiating event for systemic mediator release is the underlying ischemia to the limb or organ. This release of systemic mediators results in increased expression of adhesion molecules and neutrophil activation, resulting in overall higher levels of neutrophil-endothelial cell adhesion [11]. This process also results in increased chemotaxis of leukocytes, subsequently increased permeability of the vascular wall, and eventual cell death. The role of neutrophils in increasing vascular wall permeability was confirmed by Hernandez et al. who showed those cell lines that were neutropenic had significant protection against an ischemia reperfusion-induced microvascular injury [12].

The discussion of margination central to ischemia-reperfusion injury is important in our patient. Due to the work by Hernandez et al., it is known that neutrophil activation and subsequent vascular wall permeability is central to the development of ischemia-reperfusion injury. Our patient responded well to Clobetasol, a corticosteroid. Two important studies will further explain this response to a topical steroid. The first is a study done by Aldrighetti et al. that showed the incidence of postoperative ischemia-reperfusion injury decreased significantly with the use of preoperative steroids [13]. The second study was done by Weber et al. that showed steroids decrease the transcription of L-Selectin, an important mediator in neutrophil adhesion, causing subsequent de-margination of neutrophils [14]. 

Bullae can be a part of a wide differential but, when put in the appropriate clinical setting, can be narrowed down significantly. Several cases of edematous bullae following revascularization have been reported in the literature, with most of the literature coming from Bhushan et al. [15].

## Conclusions

Reperfusion injury has several, important, basic science concepts that must be understood, including biochemical processes and oxidant accumulation. In patients who require revascularization due to the debilitating nature of peripheral artery disease, reperfusion injury could be devastating. Understanding the pathophysiology of reperfusion injury and the subsequent increase in vascular permeability can help identify the underlying etiology of these lesions in our patient as well as those seen in the postoperative setting. Due to the similarity of edematous lesions found in reperfusion injury with those found in, but not limited to, bullous pemphigoid, cellulitis, and pemphigus vulgaris, it is important to identify the wide array of presenting symptoms in these various diseases. This discussion and review of literature should be used alongside clinical judgment to diagnose and manage patients.
